# Suppressive effects of deep balanced anesthesia on cellular immunity and protein expression: a randomized-controlled pilot study

**DOI:** 10.1186/s12871-025-02980-9

**Published:** 2025-03-17

**Authors:** Xuan Duc Nguyen, Audrey Horn, Dania Fischer, Grietje Beck, Cora C. Spannenberger, Brice Gaudilliere, Jean-Louis Horn, Hermann-Josef Thierse, Thomas Frietsch

**Affiliations:** 1 Department of Anesthesiology Perioperative and Pain Medicine, Stanford University, Stanford, CA 94305 USA; 2 TÜV, Occupational Health Services Gmbh, Hechingen, 72379 Germany; 3 Laboratory for Immunology & Proteomics, Department of Dermatology and University Medical Center Mannheim, University of Heidelberg, Mannheim, 68167 Germany; 4 Amita Lab, Department of Science, Mannheim, 68259 Germany; 5 Faculty University Medicine Mannheim, Anesthesia and Critical Care Medicine, Ruprecht Karls University Heidelberg, Theodor-Kutzer Ufer 1-3, Mannheim, 68165 Germany; 6 Department Anesthesiology, Ruprecht Karls University Heidelberg, Heidelberg, 69160 Germany; 7 Functional Proteome Analysis, German Cancer Research Center (DKFZ), Heidelberg, 69120 Germany; 8 German Federal Institute for Risk Assessment (BfR), Berlin, 10589 Germany

**Keywords:** Cellular immune response, Anesthesia depth, Lymphocyte proliferation, Monocyte proteome, NK-cells

## Abstract

**Background:**

It is questionable whether or not a short period of deep anesthesia can have long lasting effects on immune suppression.

**Methods:**

To analyze specific effects of deep anesthesia on immune modulation, a randomized-controlled, single-blinded study, monocentric, pilot-study was conducted at a level 1 orthopedic and trauma center. Inclusion criteria were patients scheduled for extended shoulder surgery with an ASA score between 1 to 3 (*n* = 186). Patients on immune modulating drugs or with immune deficits were excluded. The remaining patients were enrolled and randomized to either deep or light anesthesia (*n* = 18). Patient were randomized to receive either deep anesthesia or light anesthesia for 60 min or longer. The primary aim of the study was to compare cellular activity of T-cells, NK-cells and monocytes after anesthesia. Phagocytosis and cellular lysis activity of neutrophils and monocytes were analyzed by flow cytometry. Secondly, we analyzed anesthesia induced protein expresssion pattern in human monocytes by a standardized proteomic approach, implicating quantitative two-dimensional (2D) differential gel electrophoresis and Delta2D software analyses coupled with matrix-assisted laser desorption/ionization mass spectrometry (MALDI-MS) and Mascot analysis.

**Results:**

Anesthesia duration was 109 min in the deep anesthesia group with 81 ± 17 min of BIS < 45 and a mean BIS of 38 ± 14. The light anesthesia group received anesthesia for 111 min with 13 ± 8 min of BIS < 45 and a mean BIS 56 ± 8. Cytotoxic T-cells decreased fivefold in the light anesthesia group compared to the deep anesthesia group (-28 ± 13% vs. -6 ± 18%, respectively). The number of NK-cells (*p* = 0.0127) and regulatory T-cells (*p* = 0.0217) both dropped after deep anesthesia to almost half of the plasma level. Phagocytosis activity of neutrophils and monocytes was constant with a 67% decreased trend of intracellular lysis in monocytes (*p* = 0.0625). Quantitative proteomic analyses revealed 27 anesthesia-regulated protein spots in human monocytes, 14 of which were significantly identified by MALDI-MS, and were related to processes such as macrophage function and lymphocyte proliferation, tumor progression and apoptosis.

**Conclusions:**

Deep anesthesia inhibited immune competent defense cells (killer cells and regulatory T-cells) and had a general suppression on the phagocytic function of all circulating immune competent cells.

**Trial registration:**

Clinicaltrial.gov identifier: NCT02794896.

**Supplementary Information:**

The online version contains supplementary material available at 10.1186/s12871-025-02980-9.

## Background

An abundance of experimental data illustrates that surgical stress increases the likelihood of infection, cancer dissemination and metastasis during and after cancer surgery (for review see [1]). Emerging evidence suggests that anesthetic drugs may contribute to perioperative immunomodulation, potentially leading to immune suppression and increased susceptibility to infections in the postoperative period [2–5]. Immune suppression can impair the body's ability to mount an effective immune response against pathogens, predisposing patients to surgical site infections, sepsis, and other infectious complications. Furthermore, it has been postulated that anesthetic’s exposure during a critical perioperative window may play a role in cancer recurrence [6]. However, until recently, the immune suppression by anesthetic drugs was considered too short-lived to affect the outcome. A recent meta-analysis [7] suggested that the depth of anesthesia may influence long-term postoperative outcome. Deep anesthesia, characterized by a state of profound unconsciousness and suppression of reflexes, is often necessary during certain surgical procedures to ensure patient safety and comfort. However, studies have suggested that prolonged exposure to deep anesthesia may have adverse effects on various physiological systems, leading to an increased risk of complications and poorer outcomes following surgery. Anesthesia depth below a bispectral index (BIS) of 40 to 45 [8–11] was associated with a higher postoperative longterm mortality. Anesthesia administered for a period longer than 5 min below a BIS ≤ 40 [12] was also associated with a higher postoperative 24 month mortality. Especially the cancer related mortality was increased in these trials, unproportionally in comparison to the general public. However, it is essential to recognize that the relationship between deep anesthesia and postoperative outcomes is complex and multifactorial, influenced by various patient-specific factors, surgical procedures, anesthetic techniques, and perioperative management strategies. As such, further research is needed to elucidate the precise mechanisms underlying this association and identify strategies to optimize anesthesia management and improve surgical outcomes while minimizing the risks associated with deep anesthesia. Nevertheless, anesthesia- induced immune dysfunction could be a plausible explanation for an increased cancer related mortality [4, 13–17].

The question arises whether the maintenance of deep anesthesia suppresses the immune system for a short but relevant period of time during which cancer cells may escape the defense of immune cells, despite the restoration of many immune functions within days or weeks after surgery [18]. Furthermore, in the focus are drug specific effects since some anesthetics depress immune function more than others. Secondary, the doses needed for deeper anesthesia may affect by a longer elimination period.

The contradictory hypothesis is that deep anesthesia blocks a tumor specific immune response by reducing the long lasting memory function of specific tumor fighting cells such as the natural killer cells (NK) [19] and regulatory memory T-cells [2, 3].

A reduced activity of NK-cells correlates with the number of metastasis or tumor progression in animal models [20–27]. Since NK-cells have a memory function for tumor cells when stimulated by a combined activation with interleukin IL12, IL15 and IL18, NK-cells might induce a long lasting immune memory as cancer induced memory cells (CIMC) [28]. Additionally, NK-cells communicate with other T-and B-cells, monocytes and neutrophils. The memory function of some immune cells is indicated by the CD 127 + marker. CD127 + T-cells have a longer life span since the bondage to IL7 protects them from apoptosis [29].

The function of monocytes in the combat of malignant cells is well recognized [30], especially during early tumor stages [31]. Monocytes perform phagocytosis, secrete tumoricidal mediators, promote angiogenesis, remodel the extracellular matrix, recruit lymphocytes, and differentiate into tumor-associated macrophages and dendritic cells. Moreover, the acute response of monocytes to tumor cells initiate and sustain the longer-lasting response of lymphocyte-mediated immunity [32]. Still, the exact anesthesia dependent mechanism among the various tasks of monocytes for both pro- and antitumoral immunity is unclear. Thus, anesthesia may exert profound effects on various immune cell functions with potential consequences for patient outcome. This study investigates the effects of a short period of deep anesthesia on postoperative immune cell response. Healthy patients (ASA 1–3) undergoing elective extended shoulder surgery with continuous regional anesthesia for over an hour were randomly assigned to either a deep or light anesthesia level (BIS < 35 or < 55). Immune response was determined by phenotypic characterisation of various populations of B- and T-cells as well as neutrophil and monocyte phagocytosis activity, cellular lysis activitiy, and proteome analysis of monocytes immediately after the surgical stimulus and following a 12 weeks recovery period.

## Methods

### Ethical approval

Ethical approval for this pilot study (Ethikkommission II der Ruprecht-Karls-Universität Heidelberg 2007-061N-MA) was provided by the Ethical Committee II University Medicine Mannheim, University of Heidelberg, Germany (Chairperson Prof W. Striebel). The methodology and reporting of the study adheres to CONSORT guidelines. The trial was registered with the Clinicaltrial.gov identifier: NCT02794896 on December 1st, 2010.

### Study aim and design

The hypothesis of our pilot study was that, in contrast to light anesthesia, a longer period in deep anesthesia levels downregulates regulatory T-cells, NK-cells and inhibits activation of B-cells in healthy patients as well as the phagocytosis and oxidation function of neutrophils and monocytes. The primary aim was to measure these parameters as a change from a preoperative baseline to an early postoperative stage. The secondary aim was a search for the responsible protein mediators, a proteomic screen of involved monocyte proteins in the same interval.

Patients undergoing elective shoulder surgery were screened and consented to the randomized controlled, blinded study. Patients, surgeons and the study personell involved in data interpretation and management were blinded towards the randomly assigned computerized group allocation. The randomization schedule was generated by the SAS Procedure PROC RANDOM (SAS Institute Inc., Cary, NC). The study director informed the anesthesiology staff about the group allocation. Patients were randomized to deep vs. light general anesthesia guided using a bispectral index monitor (BIS Vista, Aspect) with BIS ≤ 45 in group 1 (deep anesthesia) or BIS ≥ 55 in group 2 (light anesthesia). The responsible anesthesiologist had to target BIS levels of 35 ± 5 in the deep anesthesia group or 55 ± 5 in the superficial anesthesia group. Anesthesia depth was recorded via USB port every minute. For group analysis, the time period spent in deep anesthesia below a BIS of 45 was compared.

Inclusion criteria for this study were ASA 1 to 3, planned minor shoulder surgery by the same experienced surgeon extending for a minimum of 60 min without preoperative sedatives, consent for an intrascalene brachial plexus block, and no contraindications for the planned anesthesia. Regional anesthesia was placed before surgery and consisted of a single injection mixture of 100 mg prilocaine, 15 to 20 ml of ropivacaine 0.5% and 75 µg clonidine followed by a laryngal mask airway (LMA) based balanced general anesthesia with propofol and fentanyl for induction and maintenance with sevoflurane (0.5 to 1.5 MAC (minimal alveolar concentration)). A second generation cephalosporine antibiotic was administered in the recovery phase (after the second blood sample was drawn at the end of surgery). Exclusion criteria were patients needing anxiolysis with preoperative administration of midazolam, preexisting severe immune deficiency (diabetes, steroid, or antihistamine medication; cancer, chemotherapy, status post transplantation, drug and/or alcohol abuse), or had recent (within one month) surgery or blood transfusion. Blood samples were taken under minimal stress prior to anesthesia induction (T0), on postoperative day 1 (T1) and at 12 weeks following hospital discharge (T2). This included the uncomplicated and smooth venipuncture by an experienced anesthesiologist in a friendly atmosphere and the use of large bore canula, minimal suction and the avoidance of too much cell activation. Postoperative care of all patients with minor shoulder surgery was standardized for nine weeks as described by institutional standards [33] characterized by 48 h immobilization in a Gilchrist-bandage, then sling shot abduction cushion for 21 days, the discharge following three to five days, removal of sutures after 10 to 12 days, early assisted mobilization with limited range of motion from week four until free range of motion in week 7, and the reintegration into daily activities and sports from nine to 12 weeks under further continuation of daily physiotherapy, moderate antiphlogistic and pain treatment as needed.

Bispectral index monitoring (BIS) occurred in all subjects from the awake state to complete recovery. Routine automated laboratory techniques determined hemoglobin concentration, leukocyte, and lymphocyte counts, in addition to the following specific immune tests. Proteomic analysis were planned for the first 3 subjects, only.

### Flow cytometric analysis

Phenotypic characterisation of lymphocytes was performed by flowcytometry as previously described [34, 35]. In brief, 100 µl EDTA blood were stirred with 10 µl of three fluorescent dyes (FITC, PE, PC5) marked antibody mixture containing CD3 (FITC), CD4 (FITC, PE), CD8 (FITC, PE), CD 16/56 (PE), CD25 (PC5), CD127 (PE), CD 56 (PE) and HLA-DR (PE). Cells were analysed after incubation periods of 15 min with the addition of 200 µl lysing solution and 300 µl PBS buffer. Cells were gated by fluorescence in a histogramm with FITC on the absciss and PE on the ordinate. The percentage of cells with the respective marker were then determined and quantified to the relation of the absolute number of lymphocytes.

Monocyte and neutrophil phagocytosis activity ^30^ were measured separately in macrophages of fresh heparinized blood using flow cytometric test kits. The complex phagocytosis process was analyzed in two parts: cellular uptake (phagocytosis activity) (Phagotest®, Orpegen Pharma GmbH, Heidelberg, Germany) [36, 37] and intracellular oxidation (respiratory burst activity) (Phagoburst®, Orpegen Pharma GmbH, Heidelberg, Germany) [38, 39] as used by our group before [40]. To test the phagocytosis, 100µL of heparinised blood was mixed in separate tubes with 20 µL of fluorescent dye (FITC) labelled and opsonized *E. coli* suspension (Phagotest®, Orpegen Pharma GmbH, Heidelberg, Germany). To analyze oxidative burst activity (Phagoburst®), unstained opsonised *E. coli* was used as test bacteria and was then compared to the ligand of proteinkinase C phorbol-12-myristat-13-acetat (PMA) (high activation) and to the bacterial peptide *N*-formyl-Meth-Leu-Phe (FMLP) (low activation). Oxidation of non-fluorescent dihydrorhodamine 123 (DHR) from the green fluorescent rhodamine 123 (R123) was used as an indicator of respiratory burst reactivity of neutrophils. In both tests (Phagotest® and Phagoburst®), the incubation period was 10 min at 37 °C. Phagocytic uptake was stopped by transferring the test tube on ice and the fluorescence of remaining extracellular *E. coli* was eliminated by the addition of a quenching solution. Red cells were lysed (lysing solution, Orpegen) and the remaining cells were washed with a stabilized phospate-buffered saline solution. A DNA staining solution containing propidium iodide was used to discriminate between diploid human cells from aggregates of bacteria or cells in the histogram. To find phagocytosing monocytes or granulocytes, a gate was set in the scattered light of the FACScan™ using an argon laser 488 nm (lin FSC vs. lin SSC). Following adjustment with the negative control (0 °C), the percentage of phagocytosing cells was counted and ingested bacteria was reflected by the mean intensity of green fluorescence from the positive cells. The manufacturer (PhagoBurst®) stated that normal values of intracelluar oxidation and phagocytotic activity (PhagoTest®) for healthy volunteers were 70–100% (monocytes) and 95–100% (neutrophils), and 65–95% (monocytes) and 95–99% (neutrophils) respectively.

### Proteomics

Isolation of human monocytes for proteomic analyses. Quantitative protein expression of monocytes was examined by isolating monocytes from heparinized blood using Ficoll-Hypaque densitiy gradient separation (Biocoll-Separation-Solution, density 1.077 g/ml; Biochrom AG, Berlin, Germany) and CD14 positive Micro Bead sorting (MACS method; isotype mouse IgG2a; QuadroMACS Separator, Miltenyi Biotec GmbH, Bergisch-Gladbach, Germany) as described by us before [41]. The numbers of monocytes in 1 ml were counted in a Neubauer chamber by light microscopy (DMIL Leica, Leica Microsytems, Wetzlar, Deutschland), and in addition the purity of monocytes selectively confirmed by FACS analysis, before sample protein concentration was determined (Protein Determination Assay with Coomassie Plus Protein Assay Reagent; Thermo-Fisher Scientific, Waltham, USA) and considered sufficient if above ≥ 1,63 µg/µl; to perform protein separation by 2D-electrophoresis.

Protein separation. Lysed monocytic proteins were separated and displayed by 2- dimensional gel electrophoresis: first dimension, isoelectric focussing, 30 µg protein on IPG stripes (pH 4–7, 7 cm; Biorad) separated on an IPGphor according to manufacturer’s instructions (GE Healthcare, Uppsala, Sweden; 30,400 Vhrs in 14 h); second dimension, separation of reduced and alkylated proteins in polyacrylamid gels (12.5%; Ettan Dalt six Gel Caster and Ettan DALTwelve System Separation Unit, GE Healthcare) for 30 min at 5 Watts and 17 Watts as described [39, 41].

Protein visualization with Flamingo stain. The gels were stained by 10 × Flamingo fluorescent gel stain 1:10 in aqua dest. according to manufacturer’s instruction (Biorad).The Fujifilm FLA-5100 imaging system was used for fluorescent analyses (Fujifilm, Düsseldorf). Gels were scanned with 50 mm slit width and 400 V PMT setting [39].

Quantitative image analysis. Quantitative analysis of spot detection and warping was done by the densitometric group comparison of the master gel (Decodon Delta 2D, Decodon, Greifswald, Deutschland) as described previously [41] The group analysis was performed as comparison of the 4 groups following anesthesia (both anesthesia depths at T1 and T2) against the control group gel (all subjects before anesthesia at T0). By comparing gels and statistically significant protein expressions taken from different samples, the later identifications of proteins for the differential molecular processes induced by anesthesia depth is possible. The Decodon software combines fast visual analysis with very reliable approaches to spot detection, matching, quantification, and statistical significance. It provides versatile means to sort, filter and analyze the quantitative spot data [42]. Most important, the user keeps control through the user interface and handy tools to interact with the algorithms if necessary (co-editing). By using this technique for this pilot study, a low number of subjects are acceptable as has been published before, e.g. by Gavin et al. [43].

To quantify the regulation of identical expressed proteins in monocytes, the “warping” process produced identified the up- or down-regulation of identical protein spots. Up-regulation was considered at a group ratio > 1.5fold, down-regulation < 0.66 fold with a significance level of the students* t*-testing of > 80% to 95% and above 95% (as provided by the Decodon software).

Preparative gels. Using two preparative gels with either 137.5 µg or 145 µg protein per 7 cm strip (per gel; first dimension) quantitative protein expression was measured by SYPRO® Ruby Protein Gel Stain (scanning by 473 nm; filter cy3; FLA5100, Fuji Film, Tokio, Japan) followed by a secondary stain for spot picking protein with sensitive Coomassie Brilliant Blue G250 (Sigma) at a wave length of 635 nm with a related filter for cy5 (FLA5100, Fuji Film).

Mass spectrometric protein identification. Picked protein spots were analyzed with mass spectrometry by tryptic peptides as described before [39, 40] and further evaluated by alignment to the SwissProt database of human proteins (Software Mascot Search, Matrix Science, Boston, Massachusetts, USA). Common amino acid sequences and their relation of known peptides were analyzed with a Mascot Search Score ≥ 54 (Matrix Science, Boston, Massachusetts USA). A score above ≥ 54 in both gels was considered significant by Mascot Search, indicating the number of consistent peptides of the identified with the matched known proteins and by the percentage coverage of sequences within the protein.

### Statistical methods

This pilot study was done to estimate the size of a group difference for the power calculation of a subsequent trial. So, the group size of this pilot study was determined by estimation of the minimal but safe numbers of observations from experience.

Data was reported as the mean ± standard deviation in the case of continuous variables and as absolute values in the case of binary factors. Demographic data and group comparisons of protein expression were compared with a Mann Whitney U-test or student’s t-test, respectively. In order to compare two groups regarding a binary factor, Chi^2^ test or Fisher’s exact test has been used. For lymphocyte proliferation, relative changes in the area under the curve from before anesthesia administration to end of recovery were compared among groups. For lymphocyte proliferation and phagocytosis activity,in order to assess the impact of the groups and the various times, a 2-way ANOVA for repeated measurements has been done using the SAS procedure SAS MIXED with the fixed factors “group” and “time” and the random factor “Patient’s ID”; Furthermore, a 1-way ANOVA for repeated measurements has been performed within each group. If the result of this ANOVA revealed to be significant, pairwise comparisons between the time points have been performed. Because of the rather small sample sizes and the character of the study as a pilot study no adjustment for multiple comparisons has been done. For selection of multiple protein spots, the used Decodon-software uses various levels of false discovery rate detection [44–46]. For analyses of protein expression levels, the means of various groups were compared by two-factor analysis of variance (ANOVA) with control of the false discovery rate (integrated in the Decodon software). Due to the low number of observations, the α-level was set to 10% for all tests regarding lymphocyte proliferation and phagocytosis activity. The significance level of all other tests was 5%. The data was analyzed by the SAS Software 9.4WIN (SAS Institute, Cary, NC, USA).

## Results

**Fig. 1:**
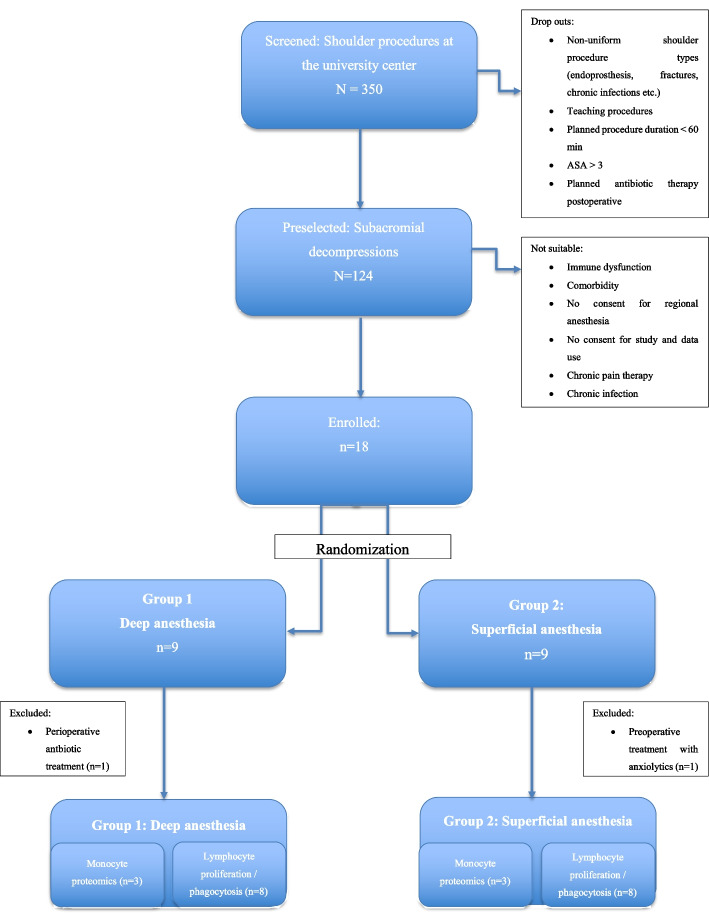
Study enrolment. A large number of consecutive patients for shoulder sugery was screened and in a second step the same surgical procedure with the same surgeon selected. The subjects should be without pre-existent immune deficiencies and comorbidities such as diabetes, bowel diseases, and ASA 1 to 2. Planned anesthesia also should be homogenously; we planned a combination of regional anesthesia (interscalene plexus block for pain control), low dose opioid induction and propofol, no muscle relaxant, and anesthesia maintenance with sevoflurance for anesthesia depth at the target level deep or superficial

The screening process included first the screening of the surgical preschedule for suitable and uniform procedures, length, surgeon, comorbidities, previous surgeries, analgetic use, ASA risk categories, and surgical follow-up plans. By a second step, the preanesthetic assessment consented for the combination of regional and (light or deep) general anesthesia as well as for study approval. From 350 screened subjects, 18 patients were eligible, were willing to consent and were enrolled in our study (Fig. 1). Sixteen patients (Eight in each group) of ASA classification 1 or 2 were enrolled and randomized in the study. The surgical procedures were minor shoulder surgical procedures of comparable invasiveness performed from the same surgeon (see detailed list of procedures in the supplementary material, Appendix).

Demographic data of both groups were very similar for age, BMI and anesthesia duration. Among the subjects under deep anesthesia, the female gender was 37.5% as opposed to light anesthesia 12.5%. (n.s.). As revealed by chart review, postoperative care and treatments were similar for both groups without differences. The first three patients in each group were allocated to additional protein expression tests (Fig. 1).

The chosen BIS target (35 and 55) produced cumulative durations of anesthesia levels below and above BIS 45 of 74.3% (deep) and 77.4% (superficial). As intended, cumulative duration of deep anesthesia was longer in group 1. Anesthesia duration was 110 min in the deep anesthesia group with 81 ± 17 min of BIS < 45 and a mean BIS of 38 ± 14 vs. 111 min for the light anesthesia with 13 ± 8 min of BIS < 45 and a mean BIS 56 ± 8 (*p* < 0.001).

In the subgroup for the protein expression tests, there was a slightly smaller difference in the cumulative deep anesthesia. Group 1 had a cumulative time 3.58 times longer at a BIS level of or below 45 than group 2. In other demographic data, there was no difference between groups (Tables 1 and 2). The planned procedure of laparoscopic shoulder surgery was switched to open decompression in one of the cases in the deep anesthesia group.

**Table 1:** All patients

Parameter or Group	Group 1 Deep anesthesia (target BIS 35 ± 5)	Group 2 Superficial anesthesia (target BIS 55 ± 5)	Significance level *p*
Number (n)	8	8	
Age (years), (median, quartiles 1, 3)	38 (27.25,52)	35 (24.5/50)	0.8915
ASA classsification	1 (*n* = 7), 2 (*n* = 1)	1 (*n* = 7), 2 (*n* = 1)	1.0000
Gender (female; male) (n,%)	3 (37.5); 5 (62.5)	1 (12.5); 7 (87.5)	0.5692
Height (cm) (median, quartiles 1, 3)	176 (172, 180)	180 (172, 185)	0.3924
Body weight (kg, (median, quartiles 1, 3)	75 (59, 86)	83 (73, 90)	0.3146
BMI (median, quartiles 1, 3)	24 (22/27)	25 (25/27)	0.3026
Surgical technique			1.0000
-endoscopic (n)	7	8	
- open (n)	1	0	
Anesthesia duration in min (median, quartiles)	109 (89, 137)	97 (81, 106)	0.2687
Cumulative anesthesia depth in min below BIS 45 (median, quartiles)	78 (68, 100)	13 (8, 20)	< 0.0001*
Length of stay (median, quartiles)	4 (3, 5)	4 (3, 4,75)	0.7941
Preoperative CRP (median, quartiles)	1.9 (1.1, 5.8)	4.6 (1.5, 6.4)	0.6600

Demographic data of all patients

Tests: for gender and surgical technique Fisher Exact test, for age, height, body weight, BMI, cumulative anesthesia depth time Students t-test, for anesthesia duration, CRP, ASA Mann Whitney U-test; ASA- American Society of Anesthesiologists Risk Score

**Table 2:** Subgroup for protein expression from monocytes

Parameter or Group	Group 1 Deep anesthesia (target BIS 35 ± 5)	Group 2 Superficial anesthesia (target BIS 55 ± 5)
Number (n)	3	3
Age (years), (median, quartiles 1, 3)	36 (26,43)	33(26,46)
ASA classification	1 (*n* = 3)	1 (*n* = 3)
Gender (female; male (n))	1; 2	1; 2
Height (cm) (median, quartiles 1, 3)	176 (172, 180)	180 (172, 185)
Body weight (kg, (median, quartiles 1, 3)	75 (59, 86)	81 (73, 90)
BMI (median, quartiles 1, 3)	27(21/27)	25 (25/26)
Surgical technique		
-endoscopic (n)	2	3
- open (n)	1	0
Anesthesia duration in min (median, quartiles)	109(87/126)	106(94/114)
Cumulative anesthesia depth in min below BIS 45 (median, quartiles)	99 (100, 83)	13 (8, 20)
Length of stay (median, quartiles)	4 (3, 5)	4 (3, 4.75)
Preoperative CRP (median, quartiles)	2.1 (1.9, 2.6)	3.4 (1.4, 5.2)

### Analysis of lymphocyte subpopulation

*Group comparison from preoperative to postoperative day (POD) 1:* Compared to group 1 (deep anesthesia), group 2 (superficial anesthesia) showed a decrease of (CD3^+^CD8^+^) cytotoxic T-cells (mean ± SD, −27.39 ± 13.1% vs. −6.04 ± 18.4, *p* = 0.021). There were no group differences in all other cell populations (Table 3).

**Table 3:** Change of Lymphocyte subpopulations from surgery to postoperative day 1 (as percentual change from baseline)

**Parameter**	**Deep anesthesia** **(target BIS 35 ± 5) (%)**	**Superficial anesthesia** **(target BIS 55 ± 5) (%)**	Significance level p (* ≤ 0.1 between groups)
Number (n)	8	8	
CD3⁺(T-cells, mean ± SD)	−4.96 ± 16.01	−11.39 ± 14.09	0.5628
CD3‾CD19⁺(B-cells, mean ± SD)	−11.91 ± 16.23	6.05 ± 32.34	0.2472
CD3⁺CD4⁺ (T-helper-cells, mean ± SD)	−3.34 ± 14.83	−3.61 ± 14.02	0.9163
CD3⁺CD8⁺ (cytotoxic T-cells, mean ± SD)	−6.04 ± 18.35	−27.39 ± 13.12	0.0209*
CD3‾CD16⁺CD56⁺ (NK-cells, mean ± SD)	−34.71 ± 33.37	−31.78 ± 36.36	1.0000
CD3⁺HLADR⁺ (activated T-cells, mean ± SD)	−9.2 ± 22.61	−23.88 ± 8.49	0.1722
CD3‾HLADR⁺ (activated B-cells, mean ± SD)	−14.89 ± 16.18	2.96 ± 21.58	0.0929*
CD127⁺ (regulatory T-Cells I, mean ± SD)	−16.29 ± 13.58	−12.16 ± 20.65	0.8480
CD25⁺ (regulatory T-cells II, mean ± SD)	−6.27 ± 25.01	−17.66 ± 16.62	0.4062

^*^Significance level ≤ 0.1 between groups

**Fig. 2:**
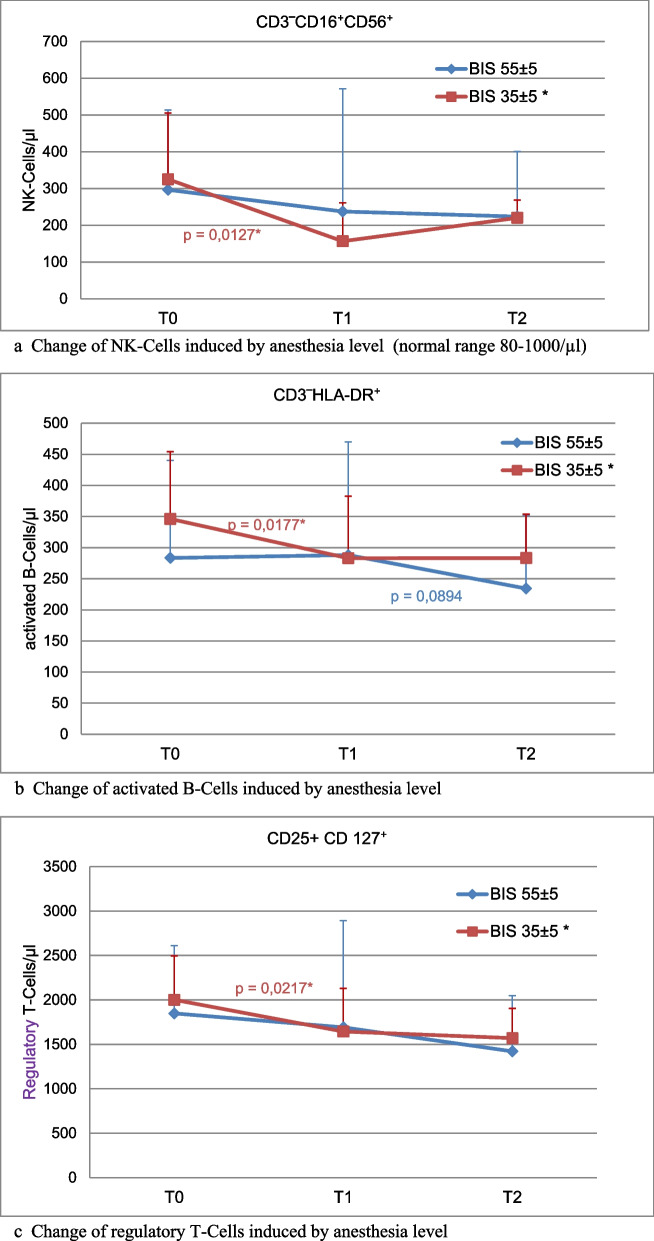
**a** Relative changes of lymphocyte subtypes over time- Natural Killer – cells. NK-cells were reduced by more than half after deep anesthesia. T0 – prior to anesthesia, T1 – immediately after anesthesia (approx. 120 min), T2 after reconvalescence (more than 3 month), BIS-bispectral index. *significant from T0 to T1, Mann Whitney U-test (**b**) Relative changes of lymphocyte subtypes over time- activated B-cells. Activated B-cells were reduced following deep anesthesia. T0 – prior to anesthesia, T1 – immediately after anesthesia (approx. 120 min), T2 after reconvalescence (3 month and more) * significant from T0 to T1 (red), from T1 to T2 (blue), Mann Whitney U-test. (**c**) Relative changes of lymphocyte subtypes over time- regulatory T-cells. Regulatory T-cells were reduced following deep anesthesia. T0 – prior to anesthesia, T1 – immediately after anesthesia (approx. 120 min), T2 after reconvalescence (3 month and more) * significant from T0 to T1, Mann Whitney U-test

*Group comparison from preoperative time to POD 2:* Compared to group 2, group 1 resulted in a decrease of natural killer cells (NK-cells CD3‾CD16⁺CD56⁺) by −51.9% vs. 15.1% (*p* = 0.013) (Fig. 2) until POD 1, but recovered within 90 days to preoperative levels. Furthermore, activated B-Cells (CD3‾HLADR⁺) and CD127 + regulatory T-cells decreased in group 1 by 18.2% (*p* = 0.0177) and 17.8% (*p* = 0.0217) on POD 1, respectively, but remained unchanged in group 2 (n.s.). The analysis of the interaction from T0 (preoperative) to T2 (90 days after surgery) resulted in a significant decrease of various lymphocyte subsets in group 1 (CD3‾CD16⁺CD56⁺ *p* = 0.0385; CD3‾HLADR⁺ *p* = 0,0083*; CD127^+^ *p* = 0.0090*).

### Phagocytosis activity of neutrophils and monocytes

The neutrophils and monocytes phagocytosis activity of test bacteria were similar after anesthesia at various depths (Phagotest n.s., not shown). After a 3 month-long recovery period, the activity levels in both groups were comparable.

**Fig. 3:**
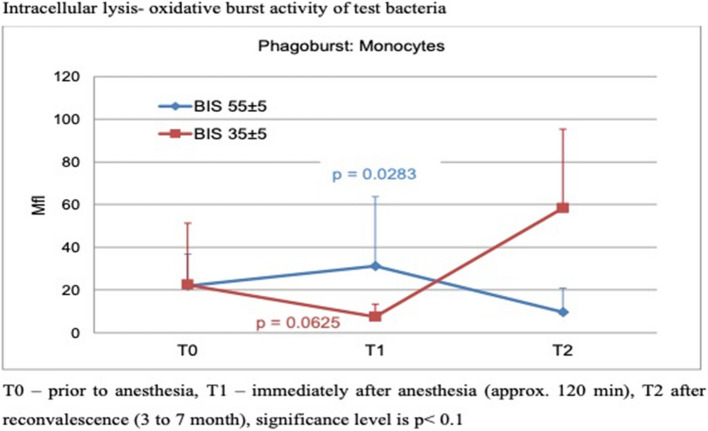
Intracellular lysis- monocytes’ oxidative burst activity of test bacteria. Neither by deep nor by superficial anesthesia, intracellular lysis was depressed or modulated to a significant difference between groups. T0 – prior to anesthesia, T1 – immediately after anesthesia (approx. 120 min), T2 after reconvalescence (3 month and more). *significant from T0 to T1, Wilcoxon signed rank test (red), **significant between groups at T1, Mann Whitney U-test

Intracellular lysis activity under various anesthesia depth was unchanged in neutrophils and monocytes. Oxidative burst in group 1 had a negative trend in neutrophils by −66.8% immediately after anesthesia as compared to an increase of + 47.8% in group 2 (Fig. 3) and in monocytes by −67% (*p* = 0.0625). The group comparison of the interaction from T0 to T2 of intracellular lysis activity of monocytes showed significance (*p* = 0.0500*).

### Protein expression of monocytes

**Fig. 4:**
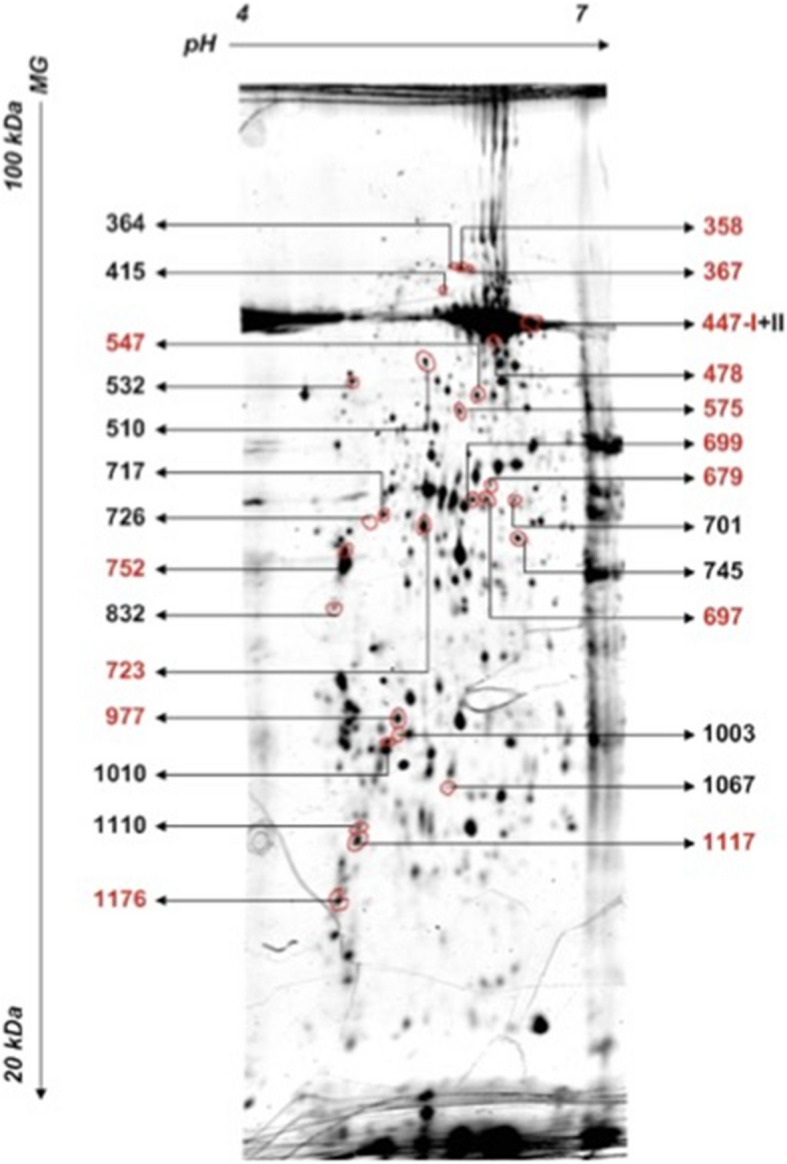
Gel spots identified (27 from 510 by Software Decodon Delta 2D, Decodon, Greifswald, Deutschland (flamingo-stain; IPG-stripes 7 cm; pH 4–7; 3 stripes pro 12,5% polyacrylamid gel (26 × 20 cm)) Dimensions (pH and molecular weight in italic numbers, kDa = 10^3^ Dalton). Red: identified by mass spectrometry

In total, 520 spots were identified by the identifying densitometric software Decodon Delta 2D [41, 47]. From all spots, only 27 were measured with significant changes between groups (Fig. 4) whereas 14 could be identified by mass spectrometry (below). Proteins were allocated in three regulation patterns- exclusive changes in the light anesthesia group (list 1), exclusive changes in the deep anesthesia group (list 2) or changed in both groups (list 3). List 1 comprised 10 upregulated proteins (547, 575, 717, 977, 723, 726, 745, 1176, 364, 532) and three downregulated proteins (1067, 1117, 752). In list 2, 12 proteins (1010, 447, 358, 367, 415, 1003, 510, 679, 701, 697, 699, 447) were up- and one (832) downregulated. In list 3, spot 1010 indicated a slight downregulation of a 0.43 fold by light anesthesia but was upregulated by deep anesthesia by 1.64 fold.

From 27 protein spots with a changed expression by anesthesia depth (Fig. 4, Table 4), 14 proteins could be identified by mass spectrometry (MALDI-TOF Ultraflex II). The known function of these proteins (Table 5) indicate a role in two major areas of interest- (1) macrophage function and lymphocyte proliferation such as a 2.21 fold decreased expression of Myoferlin, and a 1.76 fold reduced expression of RILP-like protein 1 and (2) tumor progression such as a 3.41 fold decreased expression of PHLDA-3, 1.45 fold reduction in Programmed Cell Death Protein 4 expression.

**Table 4:** Expression changes of monocytes protein spots

**Spot n°**	**Protein**	**Data base accession n°**	**Sequence covered (%)**	**Identified peptides (n)**	**Identified peptides (%)**	**Score**	**MW (da)**	**pl**
**ID-group 1**								
A447-I	Programmed cell death protein 4	sp|Q53EL6| PDCD4_HUMAN	20	8	23,5	66	52088	5,07
A697	RILP-like protein 1	sp|Q5EBL4|RIPL1_ HUMAN	29	9	31	68	47136	5,13
A723	Centromere protein K	CENPK_HUMAN	28	6	30	55	31806	4,83
B1117	Phosphatidylinositol- glycan-specific phospholipase D 2 precursor	PHLD2_HUMAN	12	7	25	58	93234	5,88
A1176	Vimentin	sp|P08670|VIME_H UMAN	25	9	23,1	64	53676	5,06
**ID-group 2 **								
B358	Calcium homeostasis endoplasmic reticulum protein	sp|Q13201|CHERP _HUMAN	9	7	41,2	60	104078	9,08
B367	Mitotic checkpoint serine/threonine- protein kinase BUB1 beta	BUB1B_HUMAN	6	6	33,3	55	120753	5,17
A478	Myoferlin (Fer-1- like protein 3)	sp|Q9NZM1| MYOF_HUMAN	8	14	37,8	55	236100	5,84
A547	Programmed cell death protein 4	sp|Q53EL6| PDCD4_HUMAN	12	5	19,2	43	52088	5,07
A575	Spliceosome RNA helicase BAT1	UAP56_HUMAN	15	5	31,3	52	49416	5,44
B575	Methionine synthase	sp|Q99707|METH_ HUMAN	12	13	25	57	141749	5,39
B699	Pleckstrin homology- like domain family A member 3	sp|Q9Y5J5P|HLA3_ HUMAN	41	7	15	64	14053	9,72
A752	Microtubule-actin cross-linking factor 1, isoforms 1/2/3/5	MACF1_HUMAN	3	15	48,4	55	623626	5,27
A/B977	Collagen alpha-3(VI) chain	sp|P12111|CO6A3_ HUMAN	6	12	52,2	65	345163	6,26
**ID-group 3 **								
B447-I	Structural maintenance of chromosomes protein 1A	sp|Q14683|SMC1A _HUMAN	11	11	30	51	143771	7,51
A679	Protein KIAA1688	K1688_HUMAN	12	9	30	54	122236	7,3
A699	Multimerin-1 precursor	sp|Q13201|MMRN1 _HUMAN	11	9	24,3	50	139276	8,25

## Discussion

For the first time, this in-vivo study illustrates the impact of deep anesthesia on a very specific immune response in immune competent patients: Deep anesthesia causes a reduction of natural killer cells, CD127 + regulatory T-cells and activated B-lymphocytes. Furthermore, deep anesthesia induced a blockade of intracellular lysis function in monocytes and a specific protein expression pattern.

**Table 5:**
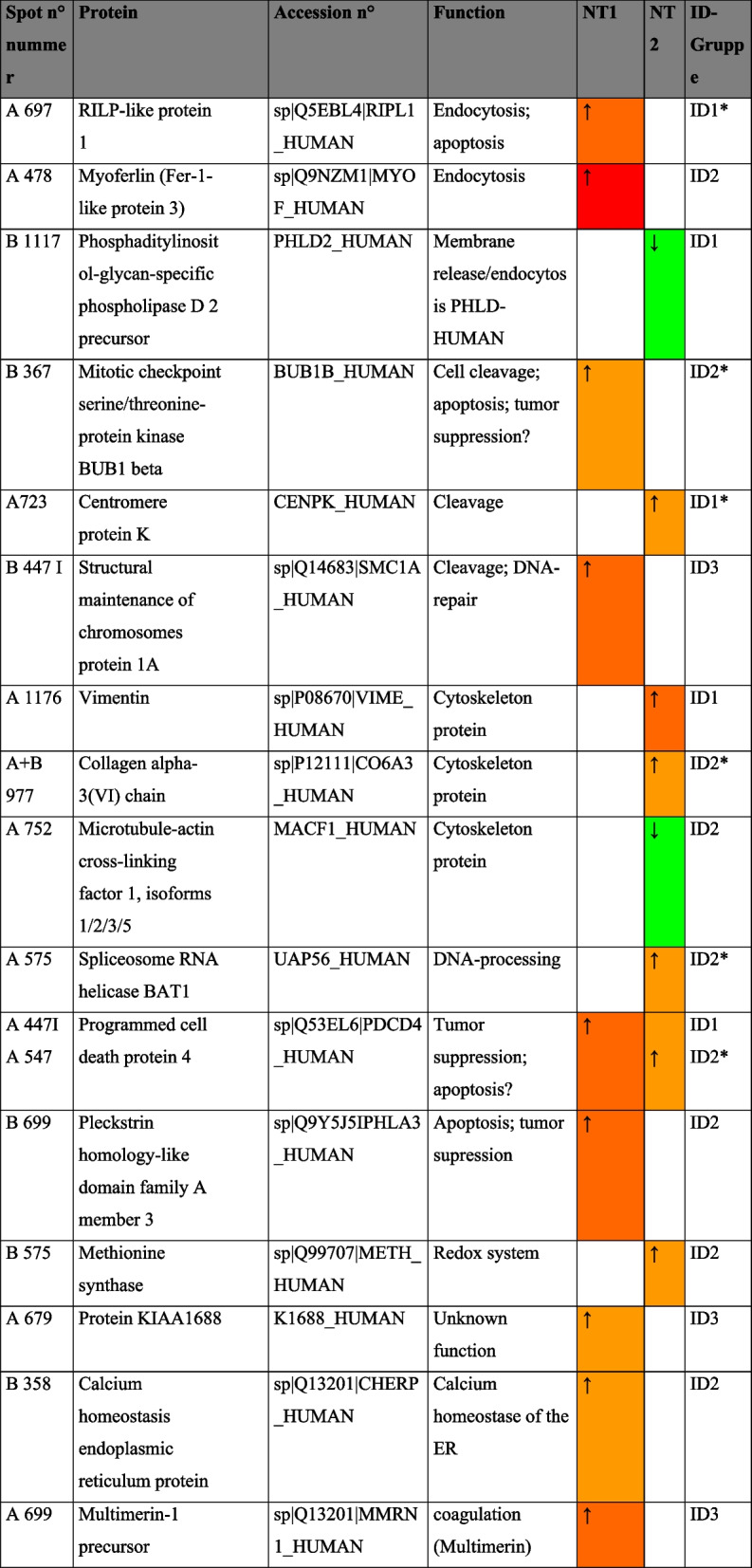
Identified proteins, names, known function and *expression in deep anesthesia group

Table 5: Proteins identified (MALDI-TOF Ultraflex II, Bruker Daltonics, Bremen, Germany; SwissProt Data base; Level of identification 95 % (*p* <0,05; Score ≥54)). A and B: two gels prepared; Spot n° as in Fig. 4, increasing within groups; MW = molecular weight, pI = iso electric point; Da = Dalton. ID groups according to score value (> 54), MW between 20 to100 kDa, pI between pH 4 to 7: Group 1—all three criteria met. Group 2—two out of three criteria met. Group 3 – only one significant score

Our pilot study is the first to identify how deep anesthesia produces a specific pattern of cellular immune response associated with a protein expression pattern in monocytes. The most considerable result of this study is the effect of deep anesthesia on specific immune competent cells. Deep anesthesia did not cause a significant suppression of (CD3^+^CD8^+^) cytotoxic T-cells but had a longer suppression of NK-cells and CD127 + regulatory T-cells > 90 days. Especially the gap between CD8^+^ T cells and CD127 + regulatory T-cells indicates profound immune dysregulation similar to those found in HIV-infected individuals as demonstrated by Paiardini et al. [48] previously. In this study, a specific pattern of lymphocyte characteristics was an effect of deep anesthesia supporting a concept previously addressed by Romee et al. [49]: Long lasting “cytokine-induced memory-like”-NK-cells are produced from high levels of the combination of IL-12, IL-15 and IL-18 [50] released from activated dendritic cells [51] and B-cells. These cells are from the adaptive immune system for the specific defense against previously recognized tumor cells. In parallel, T-cell differentiation to T_reg_-cells with memory function is complex (for review see [52]): Deep anesthesia reduces CD127^+^ T-cells- known for antiapoptotic effects in some tumors [53, 54]. In particular, the impact of deep anesthesia on NK-cells might be one of the primary factors in cancer progression and metastases [19]. Cancer itself suppresses NK-cell activity in healthy subjects [19, 55]. Suppressed NK-cell activity is associated with tumor progression for various neoplasms [56–60]. Deep anesthesia may impact cancer growth by a depression of NK cells, ultimately reducing T-cells and killer cell activities as well as a decreased production of TNFα and IFN y. Interestingly, in parallel to the immune suppressive effects of deep anesthesia, a reduction of cytotoxic cells in the group with superficial anesthesia could be observed also. This observation could be explained by temporal CD8 down-regulation in shaping the balance of invariant natural killer T (NKT)-cell subsets by modulating TCR signaling [56–60]. By the short lived suppression of CD8, a microenvironment that fosters differentiation of innate memory-like CD8 + T cells is inhibited [61]. This may support the beneficial impact upon the immune response by superficial anesthesia.

Induced by deep anesthesia, monocyte proteomics demonstrated a specific monocyte protein expression pattern. Deep anesthesia depresses apoptosis in monocytes indicated by a 3.4 fold decreased expression of a tumor suppressor protein, pleckstrin homology-like domain family A member 3 (PHLD3). This protein inhibits apoptosis by a reduced activity of AKT [62], independent of p53. Also, deep anesthesia inhibits programmed cell death protein 4 (PCDP4) expression by a 1.4-fold reduction. Apoptosis can be triggered by signals from within the cell, such as genotoxic stress, or by extrinsic signals, such as the binding of ligands to cell surface death receptors. Dysregulation of the apoptotic cell death machinery is a hallmark of cancer. Regulation of the monocyte activation and deactivation by control of apoptosis has been shown to be important in response to TNF-alpha (TNFα) mediated infection [63]. Reduced apoptosis of monocytes in anesthetized patients has been reported previously [64]. In a comparison of uniform based general anesthesia at uncontrolled depth with sevoflurane or epidural anesthesia, 6 h following anesthesia, the minor apoptotic capable monocytes were not able to produce detectable TNFα or IL-6 levels upon stimulation with LPS [64].

This study demonstrates a specific pattern of immune response associated with a longer lasting effect on cancer cells. This applies to the pattern characteristic of specific T-cells such as T_reg_-cells, and NK-cells. Deep anesthesia reduced apoptosis signal and tumor suppressor protein expression in monocytes. This indicates that a very specific immune response is provoked by deep anesthesia especially.

One limitation of our study is that an increased pharmaceutical load of anesthesia drugs (especially sevoflurane) in the deep anesthesia group was necessary but it has already been shown that deeper levels of anesthesia require more anesthetics [65]. Furthermore, in another study of our group, we demonstrated the increased drug load of anesthetics in uncontrolled anesthesia compared to BIS-controlled light anesthesia (Horn A, Abdallah Y, Knap-Czechowsky Z, Adler G, Becker P, Bruckner T, Metz M, Frietsch T. The impact of monitoring anesthesia depth on the incidence of postoperative delirium and memory in geriatric population- a monocentric randomized controlled trial, Submitted). However, a drug induced immune dysregulation might be different from a specific suppression pattern with extended duration. With or without anesthesia, the intense crosstalk between the CNS and immune system includes cytokines [66], CD4^+^ T and CD8^+^ T-cells, and monocytes. An appropriate anesthesia depth is protective from excessive production of inflammatory cytokines [67]. An “adequate anesthesia depth” however depends on secondary factors such as surgical stress and pain control. Consequently, it is a mixture of various hypnotic depths, pain control and vegetative homeostasis. In this study, surgical stress and postoperative pain was well controlled by a standardized endoscopic shoulder procedure under regional anesthesia. This way, direct superficial anesthesia in the sense of isolated light hypnosis could induce specific immune responses itself. In our setting, superficial anesthesia maintained most of lymphocyte subpopulations as well as the phagocytotic activity of neutrophils and monocytes with one exemption- the number of (CD3^+^CD8^+^) cytotoxic T-cells decreased significantly (- 27%) as opposed to the group of deep anesthesia (6%). Most likely, this can be explained by a temporal and short-lived modulation of TCR signaling. However, a definite explanation with transfer into clinical relevance of this isolated suppressive and transient effect of light anesthesia is not yet possible.

In other trials on the comparison of various levels of anesthesia depth, frequently a mean BIS value is provided for group comparison. The mean BIS value can be a source of heterogeneity in anesthetic depth, depending on the duration patients spend below BIS 45. In consequence, we used the cumulative time below a BIS of 45 to measure the impact of deep anesthesia more sustainable.

Since we conducted a small pilot study to generate a hypothesis, the results need validation in bigger study cohorts. Moreover, since we chose young and immune competent patients to identify a basic response pattern to deep anesthesia, the link to the outcome “cancer recurrence” or cancer related mortality has yet to be confirmed.

Furthermore, a uniform, but random anesthesia drug pattern was selected. It cannot be excluded that the observed immune response is induced by the chosen anesthetics (propofol, fentanyl, sevoflurane, ropivacaine, prilocaine and clonidine). However, they were used in both groups but to a lower drug dosage in the light group. The device algorithm of BIS had been validated with propofol, at discretion of the producer. BIS however reliably indicates anesthesia depth produced by sevoflurane in a similar manner with minor differences [68]. Since both drugs might have different effect on cellular immune response, the specific effects described in this study are applicable for this (common) anesthetic drug combination used only. Nevertheless, pain induced immune modulation is unlikely since effective continous regional anesthesia technique was used in both groups (as documented by equally low pain scores in both groups in the recovery period).

The used anesthesia combination in our study, regional anesthesia combined with general anesthesia, preserves NK cell counts and CD4 levels better than general anesthesia alone for abdominal cancer surgery [16]. Although various effects on immune functions by most of common anesthetics [69] have been demonstrated, we used standard of care anesthetic drugs in both groups. In general, immunomodulatory effects of anesthetics are associated with the regulation of toll-like-receptor (TLR) signalling [69]. Propofol preserves NK cell activities better than sevoflurane [70], and produces analgesia, organ protection, immunity, and anti-oxidation by GABA receptors on macrophages and monocytes (for review see [71]. In fact, many receptors on neurons as target of anesthetic drugs are also present on immune cells such as Ca/Mg ion channel protein, TLRs, integrin β2, and Ras1 protein (Rap 1), to promote degranulation of immune cells like NK cells and macrophages and consequently decrease their killing abilities and inhibit anti-tumor effects. Propofol, reduced the releases of interleukins IL-6, IL-8, and tumor necrosis factor TNF-α by inhibiting the nucleoprotein high mobility group box 1 (HMGB1) expression and NF-κB (nuclear factor kappa-light-chain-enhancer of activated B cells) activation in various cells via modulation of nc RNA (non coding RNAs) during hypoxia and ischemia–reperfusion [69]. Volatile anesthetics such as sevoflurane dose-dependent induce apoptosis and a decrease in NK cells, as well as T-cells and B lymphocytes by activating IP3 (inositol triphosphate) [72]. Furthermore, it inhibits the activation of TLR1/TLR2 to reduce neutrophil migration. Opoids and especially fentanyl have no direct effect on immune cells but on opoid receptor carrying stem cells and tumor cells which could affect various aspects such as angiogenesis and immune regulation by activating signal pathways of PI3K (phosphatidyl-inositol-3-kinase), Akt (proteinkinase B), and mTOR (mammalian target of rapamicin), and thereby promote tumor recurrence and metastasis [72]. Moreover, their use is associated with a decrease in antibody production, inhibition of cell growth and T-cell mediated adaptive responses, induction of apoptosis, decreased effector response from B lymphocytes and increased Th1 cell death and Th2 differentiation by yet unknown pathways [73]. Also, local anesthetics such as the used ropivacaine and prilocaine might not reach high plasma levels but can contribute to a not well understood effect of anesthesia on immune functions. Amide local anesthetics affect phagocytosis activity, chemotaxis, and superoxide production by macrophages and neutrophils. Clonidine depresses NK cells and lymphocytes by alpha-adrenoreceptors (alpha-ARs) on lymphocytes for the first 24 h after surgery [74–76]. By choice of the used mix of fentanyl, propofol, and sevoflurane, we tolerated a combined effect on immune response mechanisms but similar in both groups. Although we did not investigate multiple groups on various anesthetic levels to demonstrate dose dependent differences in same immune functions and cells, we favor a specific pattern of preserved immune functions by superficial anesthetic depth.

The surgical trauma varied from individuum to the next subject but was kept in a small range. A comparison of identical procedures would be scientifically ideal but is hard to achieve with real patients. However, the same experienced surgeon confirmed that the tissue trauma of the selected surgeries was comparable.

As a further lesson for the study design of a randomized trial on this topic arises the unequal distribution of gender between groups. With high certainty, gender specific immune response patterns have become more and more meaningful for our topic. The gender imbalance in this small pilot study was not intentional and reflects the randomized allocation process combined with our relatively small sample size. Along with the more balanced gender distribution in future studies, this study request results in more than two anesthesia depths for detection of quantitative “dosing-effects”, cytokine levels for acute and short acting interleukins or TNFα. They should be measured to build up on our results as it was our primary intention to study mechanisms in the adaptive immune system that might explain a long lasting effect. This should be demonstrated in more detail and shorter intervals in the days and weeks following surgery in a greater number of subjects to reach convincing statistical power. In this pilot study, the effect of deep anesthesia on immune cells only is distinguishable between anesthesia induction and POD1. The evidence of continuing specific effects for days and weeks has still to be demonstrated. Furthermore, since the function of the detected proteins is not completely elucidated, it is difficult to interpret the contribution of proteins on a specific mechanism for the hypothesized immune suppression of deep anesthesia. The proteomic discovery approach chosen did not cover all human proteins due to isoelectric focusing in the limited range from pH4 – pH7, the limitations in the second dimension e.g. for lipoproteins, and protein detection limits. Because of the technical limitations of the Ettan-Dalt system and Decodon's image analysis software, only a limited number of proteome samples per group were analyzed [40, 73]. However, the classical differential proteomic method of 2DE, coupled with modern quantitative protein expression analysis (Delta2D Software analysis; last update 2018 Handbook) and mass spectrometry identification was chosen for reasons of safety [42, 77] and of clinical relevance [78–80]. Indeed, it might seem “an old-fashioned way", however 2D-gel-electrophoresis is still a fundamental and reliable technology of proteomics research [78, 81, 82]. Thousands (in our case, hundreds, due to experimental setup) of proteins can be separated on a single gel, representing a large share of the proteins in a sample. In addition, post-translational modifications such as isoforms and phosphoproteins, e.g. from human 26S proteasome subunits, can be detected [82]. The aim of the study as to produce relevant and strictly well-matched data from a low number of pilot study subjects, the classical proteomics method was chosen due to the better validity and more experience with it ([41, 77]. Thereby, in this stage of knowledge about the effects of anesthesia depth upon the protein expression of monocytes, the necessary data and the group differences could be even better analyzed by the used classical method than with up-to-date or modern high-throughput proteomic techniques.

The results obtained here will allow to expand the dataset in a future second targeted proteomics approach with higher throughput, e.g. via typical absolute protein quantifications by parallel reaction monitoring (PRM) in an Orbitrap-based instrument or by multiple reaction monitoring (MRM)/selected reaction monitoring (SRM) [74]. Finally, the small sample size in our pilot may have impacted the observed effects of deep anesthesia.

## Conclusions

In conclusion, this pilot study examined the effects of a short period of deep anesthesia on the immune response in terms of specific inhibition of the cell count and activity of specific immune competent defense cells such as natural killer cells and regulatory T cells over a general suppression of the phagocytic function of all circulating immune competent cells. Protein expression of monocytes, specific immune effects via PHLD3 and PCDP4 might add to a hindered specific defense mechanisms by deep anesthesia.

Since the effects of a common anesthetic drug mix performed in various anesthesia depth on otherwise healthy patients with undisturbed immune functions have not been known so far, a base line as well as the effects of various anesthesia depths are getting clearer now. Our hypothesis that there are specific patterns of immune responses rather than a dose dependent suppression of the anesthetic drug load needs to be confirmed in a greater number of patients, with additional tests that demonstrate long-lasting effects and they should be performed at more and shorter intervals. The confirmation of the specific immune response to deep anesthesia in a subsequent well powered trial would not completely rule out dose dependent anesthesia effects but would immediately change clinical anesthesia practice. Due to the indication by this study that deep anesthesia may induce profound effects on various immune cell functions with potential consequences for patient outcome even in immune competent individuals, the importance of an individualized approach in the future is highlighted.

## Supplementary Information

Supplementary Material 1.

Supplementary Material 2.

Supplementary Material 3.

## Data Availability

No datasets were generated or analysed during the current study.

## Abbreviations

2DE: Two-dimensional gel electrophoresis
Akt: Proteinkinase B, a serine/threonine protein kinase
ANOVA: Analysis of variance
ASA-Score: American Society of Anesthesiologists risk score
B-cells: A type of lymphocytes
BIS: Bispectral Index
Ca: Calcium
CD: Human cellular antigens, “cluster of differentiation” molecules
CIMC: Cancer induced memory cells
CNS: Central nervous system
DNA: Deoxyribo-nucleid-acid
DR: Human Leukocyte Antigen – DR isotype
GABA: Gamma aminobutyric acid
HLA: Human leukocyte antigen
HMGB1: High mobility group box 1
IL: Interleukin
IP3: Inositol triphosphate
FACS: Fluorescence-activated cell sorter
FITC: Fluorescein isothiocyanate
LMA: Laryngal mask airway
LPS: Lipopolysaccharide solution from gram neg. bacteria
MAC: Minimal alveolar concentration
MACS: Magnetic-activated cell sorting
MALDI-MS: Matrix-assisted laser desorption/ionization mass spectrometry
Mg: Magnesium
MRM: Multiple reaction monitoring
mTOR: Mechanistic or mammalian target of rapamycin
Nc RNA: Non-coding ribonucleid acids
NF-kB: Nuclear factor kappa-light-chain-enhancer of activated B cells
NK-cells: Natural killer cells
PCDP4: Programmed cell death protein 4
POD: Postoperative day
PBS: Phosphate buffered saline
PE: Phycoerythrin
PC5: Phycoerythrin-cyanine5 conjugate
PHLD3: Pleckstrin homology-like domain family A member 3
PMA: Proteinkinase C phorbol-12-myristat-13-acetat
PRM: Parallel reaction monitoring
SRM: Selected reaction monitoring
T-cells: A type of lymphocytes
TCR: T-cell receptor
Th1 / Th2: Type 1 / 2 T-helper cells
TLR: Toll-like receptor
TNF: Tumor necrosis factor
T_reg_-cells: Regulatory T cells

## References

[1] Gottschalk A, Sharma S, Ford J, Durieux ME, Tiouririne M. Review article: the role of the perioperative period in recurrence after cancer surgery. Anesth Analg. 2010;110(6):1636–43. 20435944 10.1213/ANE.0b013e3181de0ab6

[2] Bharati SJ, Chowdhury T, Bergese SD, Ghosh S. Anesthetics impact on cancer recurrence: What do we know? J Cancer Res Ther. 2016;12(2):464–8. 27461594 10.4103/0973-1482.148670

[3] Kurosawa S, Kato M. Anesthetics, immune cells, and immune responses. J Anesth. 2008;22(3):263–77. 18685933 10.1007/s00540-008-0626-2

[4] Kim R. Anesthetic technique and cancer recurrence in oncologic surgery: unraveling the puzzle. Cancer Metastasis Rev. 2017;36(1):159–77. 27866303 10.1007/s10555-016-9647-8

[5] Wang W, Xiao J, Shen S, Wang S, Chen M, Hu Y. Emerging effect of anesthesia on post-operative tumor recurrence and metastasis. J Int Med Res. 2019;47(8):3550–8. 31296069 10.1177/0300060519861455 PMC6726782

[6] Tavare AN, Perry NJ, Benzonana LL, Takata M, Ma D. Cancer recurrence after surgery: direct and indirect effects of anesthetic agents. Int J Cancer. 2012;130(6):1237–50. 21935924 10.1002/ijc.26448

[7] Liu YH, Qiu DJ, Jia L, Tan JT, Kang JM, Xie T, Xu HM. Depth of anesthesia measured by bispectral index and postoperative mortality: A meta-analysis of observational studies. J Clin Anesth. 2019;56:119–25. 30771713 10.1016/j.jclinane.2019.01.046

[8] Lindholm ML, Träff S, Granath F, Greenwald SD, Ekbom A, Lennmarken C, Sandin RH. Mortality within 2 years after surgery in relation to low intraoperative bispectral index values and preexisting malignant disease. Anesth Analg. 2009;108(2):508–12. 19151279 10.1213/ane.0b013e31818f603c

[9] Monk TG, Saini V, Weldon BC, Sigl JC. Anesthetic management and one-year mortality after noncardiac surgery. Anesth Analg. 2005;100(1):4–10. 15616043 10.1213/01.ANE.0000147519.82841.5E

[10] Monk TG, Weldon BC. Anesthetic depth is a predictor of mortality: it’s time to take the next step. Anesthesiology. 2010;112(5):1070–2. 20418684 10.1097/ALN.0b013e3181d5e0eb

[11] Monk TG, Weldon BC. Does depth of anesthesia monitoring improve postoperative outcomes? Curr Opin Anaesthesiol. 2011;24(6):665–9. 21971395 10.1097/ACO.0b013e32834c7acf

[12] Leslie K, Myles PS, Forbes A, Chan MT. The effect of bispectral index monitoring on long-term survival in the B-aware trial. Anesth Analg. 2010;110(3):816–22. 19910621 10.1213/ANE.0b013e3181c3bfb2

[13] Ahlers O, Nachtigall I, Lenze J, Goldmann A, Schulte E, Hohne C, Fritz G, Keh D. Intraoperative thoracic epidural anaesthesia attenuates stress-induced immunosuppression in patients undergoing major abdominal surgery. Br J Anaesth. 2008;101(6):781–7. 18922851 10.1093/bja/aen287

[14] Cata JP, Bauer M, Sokari T, Ramirez MF, Mason D, Plautz G, Kurz A. Effects of surgery, general anesthesia, and perioperative epidural analgesia on the immune function of patients with non-small cell lung cancer. J Clin Anesth. 2013;25(4):255–62. 23659826 10.1016/j.jclinane.2012.12.007

[15] Margarit SC, Vasian HN, Balla E, Vesa S, Ionescu DC. The influence of total intravenous anaesthesia and isoflurane anaesthesia on plasma interleukin-6 and interleukin-10 concentrations after colorectal surgery for cancer: a randomised controlled trial. Eur J Anaesthesiol. 2014;31(12):678–84. 24614619 10.1097/EJA.0000000000000057

[16] Konstantis G, Tsaousi G, Kitsikidou E, Zacharoulis D, Pourzitaki C. The Immunomodulatory Effect of Various Anaesthetic Practices in Patients Undergoing Gastric or Colon Cancer Surgery: A Systematic Review and Meta-Analysis of Randomized Clinical Trials. J Clin Med. 2023;12(18). 10.3390/jcm12186027 PMC10531584 37762967

[17] Potocnik I, Kerin-Povsic M, Markovic-Bozic J. The influence of anaesthesia on cancer growth. Radiol Oncol. 2024;58(1):9–14. 38378027 10.2478/raon-2024-0012 PMC10878770

[18] Slade MS, Simmons RL, Yunis E, Greenberg LJ. Immunodepression after major surgery in normal patients. Surgery. 1975;78(3):363–72. 1098195

[19] Welden B, Gates G, Mallari R, Garrett N. Effects of anesthetics and analgesics on natural killer cell activity. Aana j. 2009;77(4):287–92. 19731847

[20] Beilin B, Shavit Y, Hart J, Mordashov B, Cohn S, Notti I, Bessler H. Effects of anesthesia based on large versus small doses of fentanyl on natural killer cell cytotoxicity in the perioperative period. Anesth Analg. 1996;82(3):492–7. 8623949 10.1097/00000539-199603000-00011

[21] Ben-Eliyahu S, Yirmiya R, Shavit Y, Liebeskind JC. Stress-induced suppression of natural killer cell cytotoxicity in the rat: a naltrexone-insensitive paradigm. Behav Neurosci. 1990;104(1):235–8. 2156522 10.1037//0735-7044.104.1.235

[22] Melamed R, Bar-Yosef S, Shakhar G, Shakhar K, Ben-Eliyahu S. Suppression of natural killer cell activity and promotion of tumor metastasis by ketamine, thiopental, and halothane, but not by propofol: mediating mechanisms and prophylactic measures. Anesth Analg. 2003;97(5):1331–9. 14570648 10.1213/01.ANE.0000082995.44040.07

[23] Shavit Y, Ben-Eliyahu S, Zeidel A, Beilin B. Effects of fentanyl on natural killer cell activity and on resistance to tumor metastasis in rats. Dose and timing study Neuroimmunomodulation. 2004;11(4):255–60. 15249732 10.1159/000078444

[24] Koda K, Saito N, Takiguchi N, Oda K, Nunomura M, Nakajima N. Preoperative natural killer cell activity: correlation with distant metastases in curatively research colorectal carcinomas. Int Surg. 1997;82(2):190–3. 9331851

[25] Nesterenko MV, Tilley M, Upton SJ. A simple modification of Blum’s silver stain method allows for 30 minute detection of proteins in polyacrylamide gels. J Biochem Biophys Methods. 1994;28(3):239–42. 8064118 10.1016/0165-022x(94)90020-5

[26] Pollock RE, Lotzova E, Stanford SD. Mechanism of surgical stress impairment of human perioperative natural killer cell cytotoxicity. Arch Surg. 1991;126(3):338–42. 1825598 10.1001/archsurg.1991.01410270082013

[27] Tai LH, de Souza CT, Belanger S, Ly L, Alkayyal AA, Zhang J, Rintoul JL, Ananth AA, Lam T, Breitbach CJ, et al. Preventing postoperative metastatic disease by inhibiting surgery-induced dysfunction in natural killer cells. Cancer Res. 2013;73(1):97–107. 23090117 10.1158/0008-5472.CAN-12-1993

[28] Vivier E, Tomasello E, Baratin M, Walzer T, Ugolini S. Functions of natural killer cells. Nat Immunol. 2008;9(5):503–10. 18425107 10.1038/ni1582

[29] Kaech SM, Tan JT, Wherry EJ, Konieczny BT, Surh CD, Ahmed R. Selective expression of the interleukin 7 receptor identifies effector CD8 T cells that give rise to long-lived memory cells. Nat Immunol. 2003;4(12):1191–8. 14625547 10.1038/ni1009

[30] Olingy CE, Dinh HQ, Hedrick CC. Monocyte heterogeneity and functions in cancer. J Leukoc Biol. 2019;106(2):309–22. 30776148 10.1002/JLB.4RI0818-311R PMC6658332

[31] Franklin RA, Liao W, Sarkar A, Kim MV, Bivona MR, Liu K, Pamer EG, Li MO. The cellular and molecular origin of tumor-associated macrophages. Science. 2014;344(6186):921–5. 24812208 10.1126/science.1252510 PMC4204732

[32] Williams MA, Newland AC, Kelsey SM. The potential for monocyte-mediated immunotherapy during infection and malignancy Part I: apoptosis induction and cytotoxic mechanisms. Leuk Lymphoma. 1999;34(12):1–23. 10350328 10.3109/10428199909083376

[33] Lehmann L, Frietsch T: Anesthesia for Shoulder Surgery. In: Anesthesia for Traumatology and Orthopedia. Volume 1, edn. Edited by Frietsch T, Weiler-Lorentz A. Munich: Urban & Fischer Verlag/Elsevier GmbH; 2009: 400.

[34] Nguyen XD, Dugrillon A, Beck C, Kerowgan M, Kluter H. A novel method for simultaneous analysis of specific platelet antibodies: SASPA. Br J Haematol. 2004;127(5):552–60. 15566358 10.1111/j.1365-2141.2004.05233.x

[35] Nguyen XD, Eichler H, Dugrillon A, Piechaczek C, Braun M, Kluter H. Flow cytometric analysis of T cell proliferation in a mixed lymphocyte reaction with dendritic cells. J Immunol Methods. 2003;275(1–2):57–68. 12667670 10.1016/s0022-1759(03)00002-4

[36] Bassoe CF. Flow cytometric studies on phagocyte function in bacterial infections. Acta Pathol Microbiol Immunol Scand [C]. 1984;92(3):167–71. 10.1111/j.1699-0463.1984.tb00069.x 6507103

[37] Bjerknes R, Bassoe CF. Phagocyte C3-mediated attachment and internalization: flow cytometric studies using a fluorescence quenching technique. Blut. 1984;49(4):315–23. 6435701 10.1007/BF00320205

[38] Gessler P, Nebe T, Birle A, Haas N, Kachel W. Neutrophil respiratory burst in term and preterm neonates without signs of infection and in those with increased levels of C-reactive protein. Pediatr Res. 1996;39(5):843–8. 8726239 10.1203/00006450-199605000-00017

[39] Rothe G, Valet G. Flow cytometric assays of oxidative burst activity in phagocytes. Methods Enzymol. 1994;233:539–48. 8015489 10.1016/s0076-6879(94)33059-x

[40] Frietsch T, Fessler H, Lorentz A, Kirschfink M, Waschke KF. Effect of Transfusion of Autologous Whole Blood vs. Autologous Packed Red Cells and Fresh Frozen Plasma on the Immune System of Healthy Volunteers. Anesthesiology. 1998;89:A 1333.

[41] Jakob A, Mussotter F, Ohnesorge S, Dietz L, Pardo J, Haidl ID, Thierse HJ. Immunoproteomic identification and characterization of Ni(2+)-regulated proteins implicates Ni(2+) in the induction of monocyte cell death. Cell Death Dis. 2017;8(3):e2684. 28300831 10.1038/cddis.2017.112 PMC5386519

[42] Berth M, Moser FM, Kolbe M, Bernhardt J. The state of the art in the analysis of two-dimensional gel electrophoresis images. Appl Microbiol Biotechnol. 2007;76(6):1223–43. 17713763 10.1007/s00253-007-1128-0 PMC2279157

[43] Gavin AC, Aloy P, Grandi P, Krause R, Boesche M, Marzioch M, Rau C, Jensen LJ, Bastuck S, Dümpelfeld B, et al. Proteome survey reveals modularity of the yeast cell machinery. Nature. 2006;440(7084):631–6. 16429126 10.1038/nature04532

[44] Pan W. A comparative review of statistical methods for discovering differentially expressed genes in replicated microarray experiments. Bioinformatics. 2002;18(4):546–54. 12016052 10.1093/bioinformatics/18.4.546

[45] Sandrine Dudoit S, Yang YH, Callow MJ, Speed TP. Statistical methods for identifying differentially expressed genes in replicated cDNA microarray experiments. In. www:Department of Biochemistry, Stanford University School of Medicine; 2000: 38.

[46] Alban A, David SO, Bjorkesten L, Andersson C, Sloge E, Lewis S, Currie I. A novel experimental design for comparative two-dimensional gel analysis: two-dimensional difference gel electrophoresis incorporating a pooled internal standard. Proteomics. 2003;3(1):36–44. 12548632 10.1002/pmic.200390006

[47] Decodon: ANALYZING 2D GELS-AS EASY AS POINT AND CLICK. In. www: Decodon GmBH; 2005. https://www.decodon.com/delta2d.html.

[48] Paiardini M, Cervasi B, Albrecht H, Muthukumar A, Dunham R, Gordon S, Radziewicz H, Piedimonte G, Magnani M, Montroni M, et al. Loss of CD127 expression defines an expansion of effector CD8+ T cells in HIV-infected individuals. J Immunol. 2005;174(5):2900–9. 15728501 10.4049/jimmunol.174.5.2900

[49] Romee R, Schneider SE, Leong JW, Chase JM, Keppel CR, Sullivan RP, Cooper MA, Fehniger TA. Cytokine activation induces human memory-like NK cells. Blood. 2012;120(24):4751–60. 22983442 10.1182/blood-2012-04-419283 PMC3520618

[50] Romee R, Leong JW, Fehniger TA. Utilizing cytokines to function-enable human NK cells for the immunotherapy of cancer. Scientifica. 2014;2014: 205796. 25054077 10.1155/2014/205796 PMC4099226

[51] Nizzoli G, Krietsch J, Weick A, Steinfelder S, Facciotti F, Gruarin P, Bianco A, Steckel B, Moro M, Crosti M, et al. Human CD1c+ dendritic cells secrete high levels of IL-12 and potently prime cytotoxic T-cell responses. Blood. 2013;122(6):932–42. 23794066 10.1182/blood-2013-04-495424

[52] Geginat J, Paroni M, Maglie S, Alfen JS, Kastirr I, Gruarin P, De Simone M, Pagani M, Abrignani S. Plasticity of human CD4 T cell subsets. Front Immunol. 2014;5:630. 25566245 10.3389/fimmu.2014.00630 PMC4267263

[53] Cekic C, Linden J. Adenosine A2A receptors intrinsically regulate CD8+ T cells in the tumor microenvironment. Cancer Res. 2014;74(24):7239–49. 25341542 10.1158/0008-5472.CAN-13-3581 PMC4459794

[54] Kaech SM, Ahmed R. Immunology. CD8 T cells remember with a little help. Science. 2003;300(5617):263–5. 10.1126/science.1084511 12690179

[55] Smyth MJ, Crowe NY, Hayakawa Y, Takeda K, Yagita H, Godfrey DI. NKT cells - conductors of tumor immunity? Curr Opin Immunol. 2002;14(2):165–71. 11869887 10.1016/s0952-7915(02)00316-3

[56] Buckley A, McQuaid S, Johnson P, Buggy DJ. Effect of anaesthetic technique on the natural killer cell anti-tumour activity of serum from women undergoing breast cancer surgery: a pilot study. Br J Anaesth. 2014;113(Suppl 1):i56-62. 25009196 10.1093/bja/aeu200

[57] Dong H, Zhang Y, Xi H. The effects of epidural anaesthesia and analgesia on natural killer cell cytotoxicity and cytokine response in patients with epithelial ovarian cancer undergoing radical resection. J Int Med Res. 2012;40(5):1822–9. 23206463 10.1177/030006051204000520

[58] Volk T, Schenk M, Voigt K, Tohtz S, Putzier M, Kox WJ. Postoperative epidural anesthesia preserves lymphocyte, but not monocyte, immune function after major spine surgery. Anesth Analg. 2004;98(4):1086–92. table of contents. 10.1213/01.ANE.0000104586.12700.3A 15041604

[59] Wada H, Seki S, Takahashi T, Kawarabayashi N, Higuchi H, Habu Y, Sugahara S, Kazama T. Combined spinal and general anesthesia attenuates liver metastasis by preserving TH1/TH2 cytokine balance. Anesthesiology. 2007;106(3):499–506. 17325508 10.1097/00000542-200703000-00014

[60] Yokoyama M, Itano Y, Mizobuchi S, Nakatsuka H, Kaku R, Takashima T, Hirakawa M. The effects of epidural block on the distribution of lymphocyte subsets and natural-killer cell activity in patients with and without pain. Anesth Analg. 2001;92(2):463–9. 11159252 10.1097/00000539-200102000-00035

[61] Kojo S, Ohno-Oishi M, Wada H, Nieke S, Seo W, Muroi S, Taniuchi I. Constitutive CD8 expression drives innate CD8(+) T-cell differentiation via induction of iNKT2 cells. Life Sci Alliance. 2020;3(2). 10.26508/lsa.202000642 PMC6985454 31980555

[62] Kawase T, Ohki R, Shibata T, Tsutsumi S, Kamimura N, Inazawa J, Ohta T, Ichikawa H, Aburatani H, Tashiro F, et al. PH domain-only protein PHLDA3 is a p53-regulated repressor of Akt. Cell. 2009;136(3):535–50. 19203586 10.1016/j.cell.2008.12.002

[63] Dreschers S, Gille C, Haas M, Grosse-Ophoff J, Schneider M, Leiber A, Buhring HJ, Orlikowsky TW. Infection-induced bystander-apoptosis of monocytes is TNF-alpha-mediated. PLoS ONE. 2013;8(1): e53589. 23349721 10.1371/journal.pone.0053589 PMC3547953

[64] Matsota P, Kostopanagiotou G, Kalimeris K, Pandazi A, Kotsaki A, Kontogiannopoulou S, Giamarellos-Bourboulis EJ. Transient Effects of Anesthesia on Leukocyte Apoptosis and Monocyte Cytokine Stimulation: A Clinical Study. Immunol Invest. 2018;47(4):327–34. 29412077 10.1080/08820139.2018.1435690

[65] Weber F, Walhout LC, Escher JC. The impact of Narcotrend™ EEG-guided propofol administration on the speed of recovery from pediatric procedural sedation-A randomized controlled trial. Paediatr Anaesth. 2018;28(5):443–9. 29575232 10.1111/pan.13365

[66] Fung TC, Olson CA, Hsiao EY. Interactions between the microbiota, immune and nervous systems in health and disease. Nat Neurosci. 2017;20(2):145–55. 28092661 10.1038/nn.4476 PMC6960010

[67] Hou BJ, Du Y, Gu SX, Fan J, Wang R, Deng H, Guo DX, Wang L, Wang YY. General anesthesia combined with epidural anesthesia maintaining appropriate anesthesia depth may protect excessive production of inflammatory cytokines and stress hormones in colon cancer patients during and after surgery. Medicine (Baltimore). 2019;98(30): e16610. 31348308 10.1097/MD.0000000000016610 PMC6708929

[68] Bresson J, Gayat E, Agrawal G, Chazot T, Liu N, Hausser-Haw C, Fischler M. A Randomized Controlled Trial Comparison of NeuroSENSE and Bispectral Brain Monitors During Propofol-Based Versus Sevoflurane-Based General Anesthesia. Anesth Analg. 2015;121(5):1194–201. 26489054 10.1213/ANE.0000000000000922

[69] Liu Y, Liu L, Xing W, Sun Y. Anesthetics mediated the immunomodulatory effects via regulation of TLR signaling. Int Immunopharmacol. 2021;101(Pt B): 108357. 34785143 10.1016/j.intimp.2021.108357

[70] Cho JS, Lee MH, Kim SI, Park S, Park HS, Oh E, Lee JH, Koo BN. The effects of perioperative anesthesia and analgesia on immune function in patients undergoing breast cancer resection: a prospective randomized study. Int J Med Sci. 2017;14(10):970–6. 28924368 10.7150/ijms.20064 PMC5599920

[71] Yi S, Tao X, Wang Y, Cao Q, Zhou Z, Wang S. Effects of propofol on macrophage activation and function in diseases. Front Pharmacol. 2022;13: 964771. 36059940 10.3389/fphar.2022.964771 PMC9428246

[72] Luan T, Li Y, Sun L, Xu S, Wang H, Wang J, Li C. Systemic immune effects of anesthetics and their intracellular targets in tumors. Front Med (Lausanne). 2022;9: 810189. 35966857 10.3389/fmed.2022.810189 PMC9365985

[73] Ackerman RS, Luddy KA, Icard BE, Pineiro Fernandez J, Gatenby RA, Muncey AR. The effects of anesthetics and perioperative medications on immune function: a narrative review. Anesth Analg. 2021;133(3):676–89. 34100781 10.1213/ANE.0000000000005607

[74] Bao JY, Huang Y, Wang F, Peng YP, Qiu YH. Expression of alpha-AR subtypes in T lymphocytes and role of the alpha-ARs in mediating modulation of T cell function. NeuroImmunoModulation. 2007;14(6):344–53. 18463421 10.1159/000129670

[75] Forget P, Collet V, Lavand’homme P, De Kock M. Does analgesia and condition influence immunity after surgery? Effects of fentanyl, ketamine and clonidine on natural killer activity at different ages. Eur J Anaesthesiol. 2010;27(3):233–40. 19487949 10.1097/EJA.0b013e32832d540e

[76] von Dossow V, Baehr N, Moshirzadeh M, von Heymann C, Braun JP, Hein OV, Sander M, Wernecke KD, Konertz W, Spies CD. Clonidine attenuated early proinflammatory response in T-cell subsets after cardiac surgery. Anesth Analg. 2006;103(4):809–14. 17000786 10.1213/01.ane.0000237308.28739.d8

[77] Dietz L, Esser PR, Schmucker SS, Goette I, Richter A, Schnölzer M, Martin SF, Thierse HJ. Tracking human contact allergens: from mass spectrometric identification of peptide-bound reactive small chemicals to chemical-specific naive human T-cell priming. Toxicol Sci. 2010;117(2):336–47. 20631061 10.1093/toxsci/kfq209

[78] Raza SK, Saleem M, Shamsi T, Choudhary MI, Atta Ur R, Musharraf SG. 5D proteomic approach for the biomarker search in plasma: Acute myeloid leukaemia as a case study. Sci Rep. 2017;7(1):16440. 29180721 10.1038/s41598-017-16699-2 PMC5703949

[79] Sousa P, Silva L, Luís C, Câmara JS, Perestrelo R: MALDI-TOF MS: A Promising Analytical Approach to Cancer Diagnostics and Monitoring. Separations 2023, 10(8).

[80] Vélez P, Ocaranza-Sánchez R, López-Otero D, Grigorian-Shamagian L, Rosa I, Bravo SB, González-Juanatey JR, García Á. 2D-DIGE-based proteomic analysis of intracoronary versus peripheral arterial blood platelets from acute myocardial infarction patients: Upregulation of platelet activation biomarkers at the culprit site. Proteomics Clin Appl. 2016;10(8):851–8. 27095521 10.1002/prca.201500120

[81] Herzog R, Wagner A, Wrettos G, Stampf K, Bromberger S, Sperl E, Kratochwill K. Improved alignment and quantification of protein signals in two-dimensional Western blotting. J Proteome Res. 2020;19(6):2379–90. 32402202 10.1021/acs.jproteome.0c00061

[82] Hirano H, Shirakawa J. Recent developments in Phos-tag electrophoresis for the analysis of phosphoproteins in proteomics. Expert Rev Proteomics. 2022;19(2):103–14. 35285370 10.1080/14789450.2022.2052850

