# Supplementary figures and images for: Suppressive effects of deep balanced anesthesia on cellular immunity and protein expression: a randomized-controlled pilot study
Source: BMC Anesthesiol. 2025 Mar 17;25:129. doi: 10.1186/s12871-025-02980-9 (PMC11912595; doi:10.1186/s12871-025-02980-9)

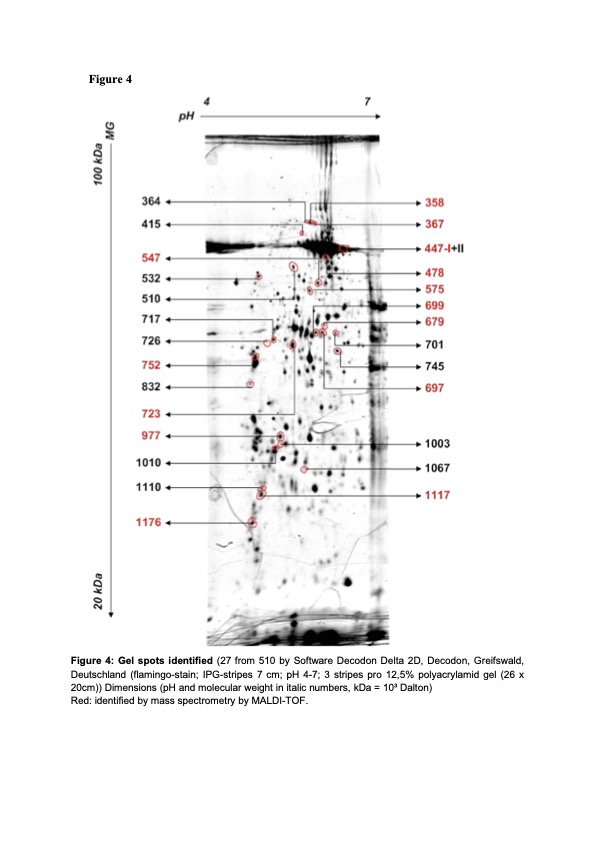

Supplement: Supplementary file 1 — Supplementary Material 1. [file 12871_2025_2980_MOESM1_ESM.jpg]
