# Supplementary material for: Suppressive effects of deep balanced anesthesia on cellular immunity and protein expression: a randomized-controlled pilot study
Source: BMC Anesthesiol. 2025 Mar 17;25:129. doi: 10.1186/s12871-025-02980-9 (PMC11912595; doi:10.1186/s12871-025-02980-9)
Supplement: Supplementary file 2 — Supplementary Material 2. [file 12871_2025_2980_MOESM2_ESM.docx]

# Appendix

## Anesthesia depth of subjects

**Figure 1 Anesthesia depth according to BIS-Monitoring of all study patients**

The BIS values of all patients during their procedures (n=16)

Group 1-Deep Anesthesia- red, Group 2-Superficial Anesthesia-green

Note that BIS monitoring for statistics of group comparison of average BIS and cumulative time periods below the threshold of BIS 45 was extracted of minute by minute recordings of each subject.

**Figure 2 Anesthesia depth /Bis-Recordings of the subgroup for additional proteomics**

## Anesthetic Consumption

## Table 1- Anesthestic Drug and Fluid Consumption

| **Group** | **1-deep** | **1. quartile** | **3. quartile** | **2-superficial** | **1. quartile** | **3. quartile** |
| --- | --- | --- | --- | --- | --- | --- |
| Vasopressor ml* (median, quartiles) | 0,75 | 0,0625 | 1,375 | 0 | 0 | 0,375 |
| Sevoflurane** ml | 11,66 | 10,41 | 15,15 | 3,71 | 3,51 | 6,92 |
| Fentanyl | 0,05 | 0,05 | 0,0875 | 0,05 | 0,05 | 0,1 |
| Propofol | 200 | 200 | 342,5 | 200 | 200 | 200 |
| Fluid*** | 575 | 325 | 700 | 400 | 287,5 | 500 |

- *As vasopressor a mixture of theodrenaline and caffedrine (AKRINOR®) is used in Germany.
- **Sevoflurane consumption is calculated as explained below
- *** A balanced crystalloid solution only is administered.
- Not mentioned are the uniform administered local anesthetics (see Methods)

Sevoflurane dose was calculated by the equation Con$=$ $\frac{FGF*Vol\%/t}{k*10000}$

FGF = Carrier gas flow in ml/min (carrier gas was oxygen/air 0.5)

Vol% = Vapor/ endtidal concentration in %

t = anesthesia duration in min

*k* = constant (for sevoflurane 186, i.E. 1 ml liquid is evapurated in 186 ml vapor)

Con = Consumption in ml

## Surgical Procedures

## Table 2- Surgical procedures for the groups

| **Group 1- deep anesthesia** |  |
| --- | --- |
| **Diagnosis** | **Procedure** |
| Bankart Lesion | Stabilisation |
| SLAP leson, rotary cuff rupture | Slap repair, suture |
| Ruptured rotary cuff and biceps tendon | Subacromial decompression and cuff repair |
| Bankart-Lesion, SLAP II | Slap repair, suture |
| SLAP IV Lesion, instability | Repair, stabilisation |
| Ruptured Supraspinatus Tendon | Subacromial decompression and tendon suture |
| Ruptured rotary cuff , subacromial impingement | Subacromial decompression and cuff repair |
| Bankart Lesion | Stabilisation |
| Group 2- superficial anesthesia |  |
| **Diagnosis** | **Procedure** |
| SLAP leson, rotary cuff rupture | Slap repair, rotary cuff repair |
| Ruptured rotary cuff and biceps tendon | Subacromial decompression, tendon suture and cuff repair |
| Bankart-Lesion, SLAP II | Labrum fixation, SLAP Repair |
| Ruptured rotary cuff and biceps tendon, subacromial impingement | Suture Tendons of suprasoinatus, long biceps tendon and subacromial decompression |
| Labrum lesion | Labrum fixation |
| Tendinitis calcarea | Resection |
| Bankart-Lesion | Stabilisation |
| Labrum lesion | Labrum fixation |

SLAP- superior labrum from anterior to posterior

## Additional Statistics for the Data in Figures 2 to 3

Two-sided-ANOVA results:

| Figure | Parameter | 2-way ANOVA | 1-way-ANOVA BIS 55±5 | 1-way-ANOVA BIS 35±5 |
| --- | --- | --- | --- | --- |
| 2a | CD3-CD16+CD56+ | group: p = 0.8949 time: p = 0.0684 | p = 0.7308 | p = 0.0176  T0-T1: p = 0.0062 T0-T2: p = 0.0438  T1-T2: p = 0.3381 |
| 2b | CD3-HLA-DR+ | group: p = 0.6089 time: p = 0.0171 | p = 0.1676 | p = 0.0159  T0-T1: p = 0.0177 T0-T2: p = 0.0083  T1-T2: p = 0.5894 |
| 2c | CD25+CD 127+ | group: p = 0.6916 time: p = 0.1121 | p = 0.6592 | p = 0.0187  T0-T1: p = 0.0217 T0-T2: p = 0.0090  T1-T2: p = 0.5292 |

| Figure | Parameter | Group Comparisons with U tests | BIS 1 | BIS 2 |
| --- | --- | --- | --- | --- |
| 3 | Phagoburst Monocytes | T0: p = 0.5476 T1: p = 0.0317  T2: p = 0.0635 | Friedman:  p= 0.2466 | Friedman:  p= 0.0250  T0-T1: p = 0.0253  T0-T2: p = 0.3173  T1-T2: p = 0.0455 |

As the parameter „Phagoburst Monocytes” doesn’t seem to be normally distributed and because of the extremely small sample sizes, we used nonparametric tests in order to compare the groups and investigate changes over time. Exact Mann Whitney U test revealed no significant difference between the BIS groups 1 and 2 at T0 (p = 0.5476). However, the difference was significant at T1 (p = 0.0317) and slightly failed significance at T2 p = 0.0635).

Using Friedman test (a nonparametric test for repeated measurements), no changes over time were found for BIS 1 (p = 0.2466). However, Friedman’s test result was significant for BIS 2 (p = 0.0250). Pairwise comparisons for BIS 2 revealed significant differences between T0 and T1 (p = 0.0253) and between T1 and T2 (p = 0.0455), but not between T1 and T2 (p = 0.3173).

## Vital Signs of Study Subjects
